# CSM-Potential: mapping protein interactions and biological ligands in 3D space using geometric deep learning

**DOI:** 10.1093/nar/gkac381

**Published:** 2022-05-24

**Authors:** Carlos H M Rodrigues, David B Ascher

**Affiliations:** Computational Biology and Clinical Informatics, Baker Heart and Diabetes Institute, Melbourne, Victoria, Australia; School of Chemistry and Molecular Biosciences, University of Queensland, Brisbane, Queensland, Australia; Computational Biology and Clinical Informatics, Baker Heart and Diabetes Institute, Melbourne, Victoria, Australia; School of Chemistry and Molecular Biosciences, University of Queensland, Brisbane, Queensland, Australia

## Abstract

Recent advances in protein structural modelling have enabled the accurate prediction of the holo 3D structures of almost any protein, however protein function is intrinsically linked to the interactions it makes. While a number of computational approaches have been proposed to explore potential biological interactions, they have been limited to specific interactions, and have not been readily accessible for non-experts or use in bioinformatics pipelines. Here we present CSM-Potential, a geometric deep learning approach to identify regions of a protein surface that are likely to mediate protein-protein and protein–ligand interactions in order to provide a link between 3D structure and biological function. Our method has shown robust performance, outperforming existing methods for both predictive tasks. By assessing the performance of CSM-Potential on independent blind tests, we show that our method was able to achieve ROC AUC values of up to 0.81 for the identification of potential protein-protein binding sites, and up to 0.96 accuracy on biological ligand classification. Our method is freely available as a user-friendly and easy-to-use web server and API at http://biosig.unimelb.edu.au/csm_potential.

## Graphical Abstract

**Graphical Abstract:**
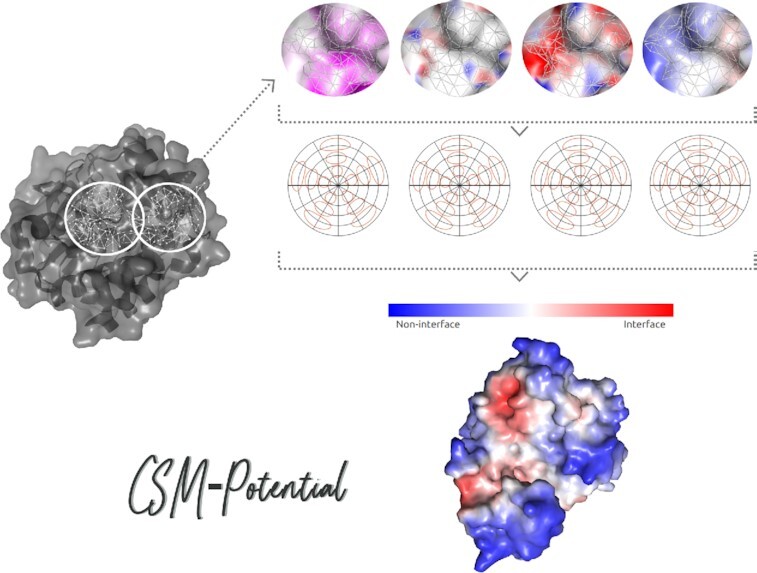
CSM-Potential.

## INTRODUCTION

Recent breakthroughs in protein structure prediction by AlphaFold (1,2) and RosettaFold (3), have led to a large proportion of the entire proteome for many organisms, now available in the AlphaFold database (4). These structures, however, lack crucial interactions, which are important for understanding protein function. Consequently, the availability of structural data has prompted an increasing demand for tools that can use these 3D models to help identify key biologically important interacting regions, which is crucial for a better understanding of their biological functions.

Significant efforts have been made towards identification of interacting sites on proteins. For prediction of PPI binding sites previous methods have ranged from those using only information about the protein sequence as input (5,6), to more complex methods, which use a combination of outputs from neural network architectures in combination with changes in relative solvent accessibility upon complexation in a consensus prediction (7). Other approaches have focused on identification of binding sites more likely to participate in interactions with ligands by exploring structural features for classification of pocket regions (8–10), representing an attractive opportunity for elucidating the mechanisms by which these compounds interact with proteins and potentially leading to the design of more safe and effective drugs. More recently, a geometric deep learning framework has been proposed (11) to extract interaction fingerprints from protein surfaces which may then be applied to tasks such as identification of PPI binding sites and binding pocket classification. Despite the diversity of methods currently available, in general these have been developed solely for exploring a specific interaction type, are computationally intensive, and not user friendly.

**Figure 1.**
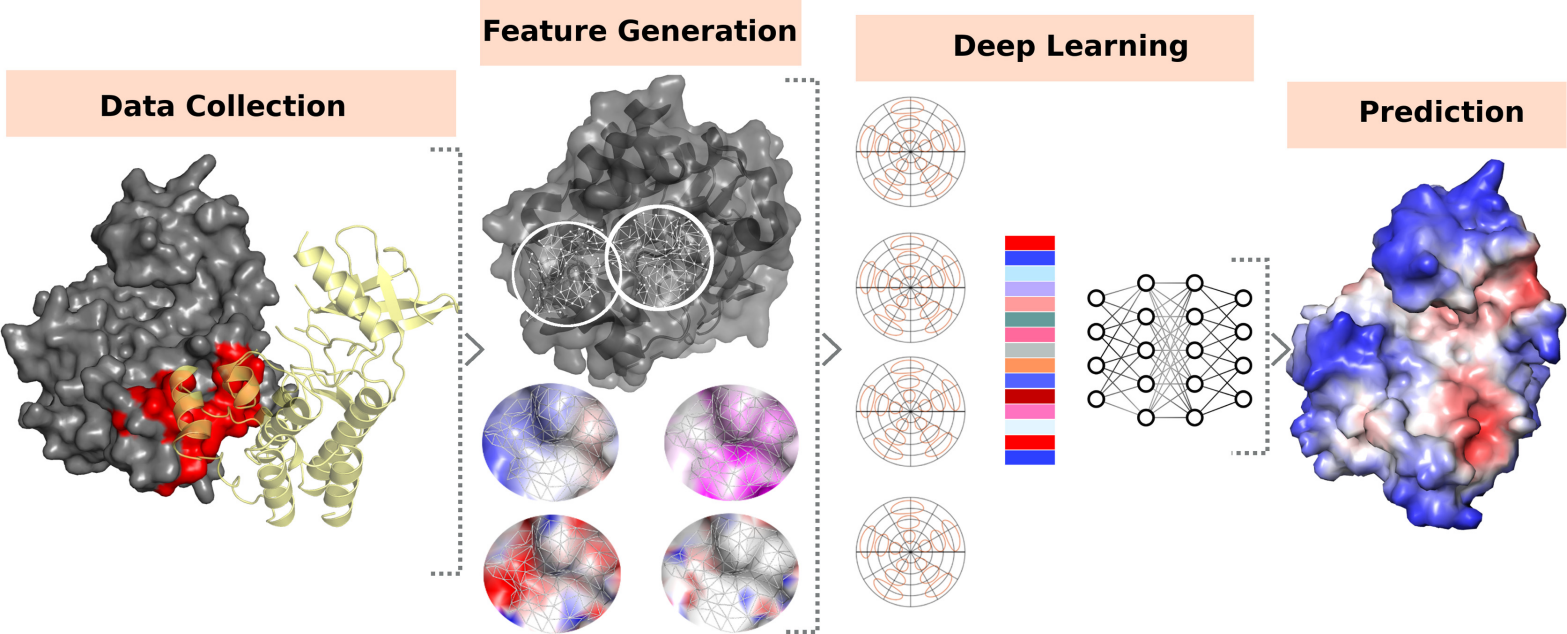
CSM-Potential methodology workflow. Experimental data on PPIs and biological ligands were extracted from the curated databases followed by interface identification based on the 3D structure. These were then used to generate molecular surfaces from which geometrical and physicochemical properties are calculated. Protein surfaces were then decomposed into overlapping patches based on a geodesic radius of 12 Å, and used in combination with features calculated on the previous step to generate embeddings from learnable Gaussian Kernels. Finally, neural networks were trained to predict PPI binding sites and to classify biological ligand binding sites.

In this study, we present CSM-Potential, a geometric deep learning approach to identify areas of a protein surface that are likely to participate in protein-protein interactions (PPIs) and protein–ligand interactions (Figure 1). Our method is implemented as an easy-to-use and freely available webserver and an Application Programming Interface (API) to facilitate integration with existing bioinformatics pipelines at http://biosig.unimelb.edu.au/csm_potential.

## MATERIALS AND METHODS

### Data sets

Experimentally characterised structures of PPI complexes were extracted from the PRISM database of non-redundant PPIs (12) for a total of 8,466 proteins. In addition, 3,536 transient interactions were taken from PDBBind (13), SabDab antibody:antigen database (14) and the ZDock benchmark set (15). Proteins were then clustered at a 30% sequence identity using CD-HIT (16) and one representative member from each cluster was chosen, resulting in 3,362 unique proteins. A pairwise matrix of all TM scores for these proteins was then computed followed by hierarchical clustering, via scikit-learn AgglomerativeClustering method, to split our dataset into training and test sets with 3004 and 358 proteins, respectively.

The database used in this work for biological ligand classification was compiled based on all structures cofactor-binding proteins available in the Protein Data Bank (PDB) (17) (accessed on 16 October 2018), where any of the following seven chemical compounds was present: Adenosine diphosphate (ADP), Coenzyme A (CoA), Flavin adenine dinucleotide (FAD), heme (HEM), Nicotinamide adenine dinucleotide (NAD), Nicotinamide adenine dinucleotide phosphate (NADP) or S-adenosyl methionine (SAM). These have been selected due to their large of structure availability in the PDB and for comparison purposes. Details for each ligand, including their chemical structure, are summarised in Table S1 and Figure S1 in the Supplementary data. This resulted in 1,853 ADP structures, 490 CoA, 2,020 FAD, 4,448 HEM, 1,269 NAD, 1,212 NAP and 393 SAM. Proteins were then clustered based on their sequence identity according to the PDB pre-computed sequence clusters, where two proteins were considered to be similar (near-identical) if the associated clusters of both proteins were the same. The final dataset used for biological ligand classification comprises 1468 structures, which were randomly split into training (72%), validation (8%) and testing sets (20%).

The approach used for curating structures for both predictive tasks, PPI binding site prediction and biological ligand classification, follows the protocol described in previous work (11).

### Geometric deep learning neural network

In this study, we apply geometric deep learning to the molecular surface of proteins in order to identify regions more likely to participate in interaction with other proteins and with biologically relevant ligands. For both predictive tasks, we trained end-to-end neural networks using the MaSIF framework (11), which decomposes protein surfaces into overlapping patches based on a geodesic radius, and then uses these in combination with geometric and chemical features to generate embeddings from learnable Gaussian Kernels (18). Here, we expanded this approach by capturing a larger geodesic radius size for patch extraction of 12 Å, and combining this with our well established graph-based signatures (19), which have been extensively applied to investigate the role of genetic mutations on protein function (20–22) and small molecule toxicity (23). These are calculated directly from the protein structure for each residue at the surface and then used to extract distance patterns between atoms characterised by their pharmacophores and compiled in signatures as cumulative distributions.

For the predictive model aiming to identify PPI interaction sites, as the number of non-interface points was usually much larger than the number of interface points, during training our neural network, a random number of non-interface points was selected until an equal number of positive and negative samples was achieved.

### Definition of interacting interfaces

PPI interacting interface was defined based on the change in solvent accessible surface area (SASA) on interaction. Here, we consider as part of the interface the region of the surface that becomes inaccessible to solvent molecules upon complex formation. This was done by comparing the difference in SASA, at the residue level, between the individual protomers (unbound protein) and within the complex as follow (24):

$$\begin{equation*}\Delta SASA\ = \ SAS{A_A}\ + \ SAS{A_B}\ - SAS{A_{AB}}\end{equation*}$$

where *A* and *B* are two proteins participating in a pairwise interaction.

Residues at the surface of the unbound partners which were not at the surface on the bound complex (solvent inaccessible) were then defined as being part of the PPI interface.

For our final dataset of protein–ligand interactions and following previous work (11), after surface and patch generation, if the center point of a patch was less than 3 Å from an atom any of the seven ligands, the patch was labeled as part of the binding pocket of the corresponding ligand.

## WEBSERVER

We have implemented CSM-Potential as a user-friendly and freely available web server (http://biosig.unimelb.edu.au/csm_potential). The server back end is developed using Python via the Flask Framework (version 1.0.2), while the front end was built using Materialize framework version 1.0.0. The web server is hosted on a Linux Server running Apache2.

### Input

CSM-Potential can be used in two different ways: for the identification of PPI sites or for the biological classification of pockets on a protein structure. In both cases, users are required to provide a protein structure by either uploading a file in PDB format or by selecting a structure directly from the PDB or AlphaFold databases using the auto-complete input field (Supplementary Figure S2A). By default, CSM-Potential will run predictions on the whole input structure, however, users may indicate specific monomers to be automatically extracted before running the predictions for structures where multiple proteins are present. Furthermore, the biological classification of pockets option requires one extra step before running the predictions, where the user must choose from a list of pockets identified using Ghecom (25) (Supplementary Figure S3A). Finally, for both options, users may provide an email address, which will be later used for notifying the user about the job's results.

### Output

For prediction of PPI binding sites (Supplementary Figure S2B), CSM-Potential summarises interface scores for each residue on the input protein structure at a sequence level using the FeatureViewer component (26). In addition, predicted scores are mapped onto the input 3D structure and displayed in an interactive viewer using NGLviewer molecular graphics library (27). Finally, users can download their input protein structure with the predictions annotated on the *b*-factor column.

For biological ligand classification (Supplementary Figure S3B), the results page shows the input 3D structure with the selected pocket region highlighted by default using NGLviewer and a downloadable table summarising the prediction score for all seven different biological ligands. For each ligand, the predicted score represents the likelihood of binding to the selected pocket. For a given ligand, additional information, such as molecular weight and number of hydrogen acceptors and donors, can be accessed via the Details button on the results table, as well as the ligand depiction built via SmilesDrawer (28) based on its canonical SMILES.

## VALIDATION

### Performance on training

We evaluated the performance of CSM-Potential to predict PPI binding sites on two different types of cross-validations on our training set. For each cross-validation type, we repeated the experiments five times and reported mean values for each evaluation metric. First we randomly selected 80% of our data for training and the remaining 20% for testing. Performance is reported in terms of ROC AUC values, which were calculated for each protein in the testing set and overall performance is reported after averaging values for all entries. Here, our method achieved an overall ROC AUC of 0.82 on average. Using a similar setup, but varying the proportion of data split between training and testing sets to 50% each, the performance of our approach remained consistent with ROC AUC of 0.79. Additional evaluation metrics, including Matthews correlation coefficient (MCC) and *F*1 score, have been summarised in Supplementary Table S2, corroborating the robustness of our trained model on CV1 and CV2. The final predictive model was then built using all entries in the training set and evaluated on a blind test set.

To train a predictive model to explore biological ligand binding sites, here we used the training and validation set described in the Materials and Methods section. Performance on the validation set was assessed at each epoch (one forward and backward pass of all entries available in the training set) and used to select the best network after each epoch. Finally, we sampled each pocket 100 times and averaged the resulting 100 predictions to obtain the final prediction, similar to previous work. At the end of our training procedure, our approach achieved a balanced accuracy of up to 0.75 and ROC AUC of 0.87.

### Blind test

**Figure 2.**
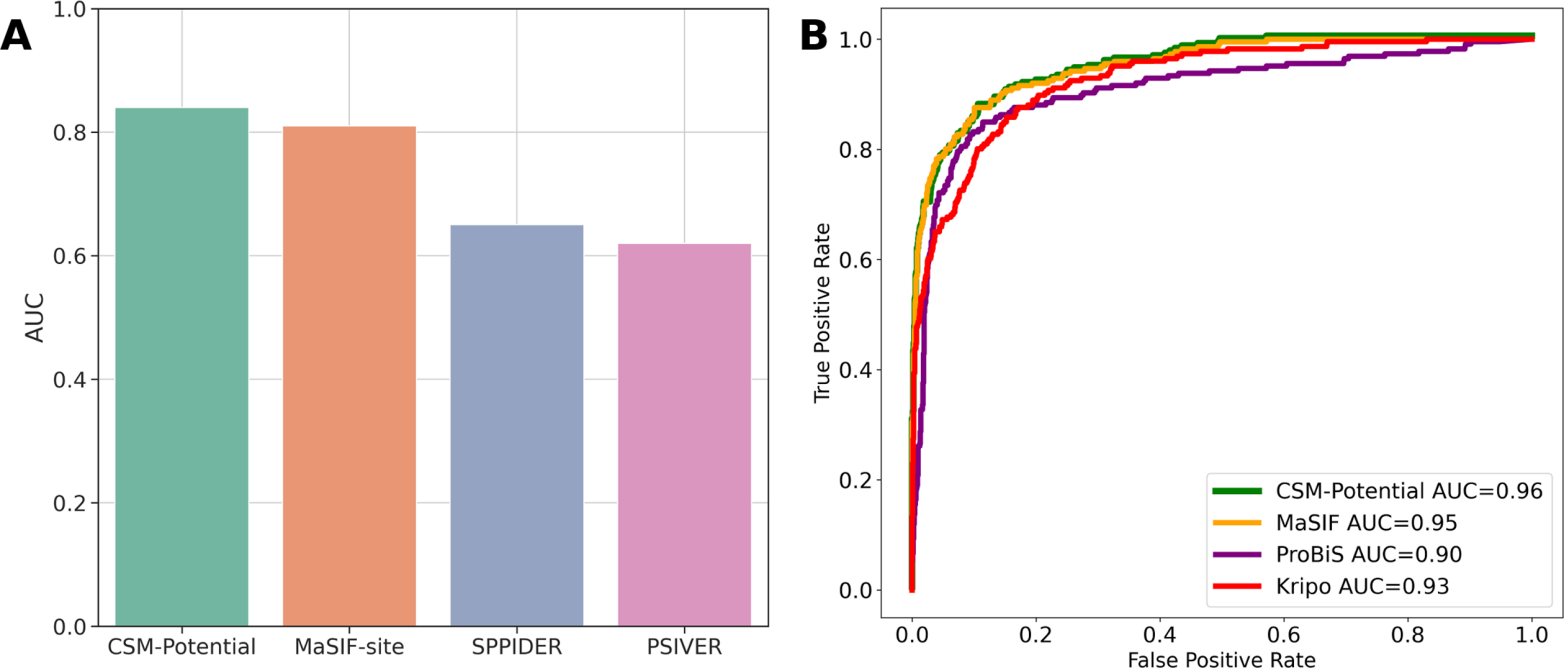
Performance comparison of CSM-Potential with alternative methods on both predictive tasks: identification of PPI binding sites and biological ligands binding sites classification. Panel **A** shows overall median ROC AUC values per protein on predictive models for the identification of PPI binding sites, while Panel **B** summarises performance in terms of ROC curves.

Here, we compared the results of our PPI binding site predictive model with those reported for MaSIF-site (11) on all 358 entries of our blind-test and three subsets of PPI interactions: transient (59), and large (74) and small (74) hydrophobic interactions. For comparison purposes, results are presented in terms of median ROC AUC per protein. Overall, CSM-Potential achieved a median ROC AUC of 0.90, while MaSIF-site achieved 0.87 ROC AUC. For the three subsets of PPI interactions, CSM-Potential outperformed MaSIF-site for all cases, achieving ROC AUC values ranging from 0.84 to 0.93 (Supplementary Figure S4). Furthermore, here we compared the results of our method with MaSIF-site, SPPIDER (7) and PSIVER (5) on a subset of 53 single-chain transient interactions. SPPIDER AND PSIVER have been previously shown to have top performance in a critical study assessing the robustness of computational predictors of PPI interfaces (29). Here, CSM-Potential achieved the highest performance over all other methods with a ROC AUC of 0.84, while MaSIF-site, SPPIDER and PSIVER achieved 0.81, 0.65 and 0.62, respectively (Figure 2A). Additional evaluation metrics (as median per protein) are shown in Table 1, showing that CSM-Potential and MaSIF-site have a more balanced prediction when differentiating between interface and non-interface residues.

**Table 1.** Performance comparison for PPI binding site prediction on a non-redundant blind test

Method	AUC	MCC	*F*1	Sensitivity	Specificity
CSM-Potential	0.84	0.23	0.24	0.78	0.75
MaSIF-site	0.81	0.21	0.20	0.75	0.73
SPPIDER	0.65	0.11	0.19	0.25	0.86
PSIVER	0.61	0.07	0.43	0.43	0.67

For assessing the performance of our predictive model for the classification of biological ligands binding sites, we first investigated how accurate CSM-Potential performed on the blind-test for each specific ligand separately (summarised in Supplementary Figure S5). Accuracies varied from 0.56 for pockets associated with SAM to 0.96 for pockets where HEM is bound. The former may be related to the small number of entries for SAM in our training set (only 23 cofactor-binding proteins), while the latter, in addition to having the highest number of entries available for training of the predictive model (57 cofactor-binding proteins), it also shows a more unique chemical structure when compared with the other ligands (Supplementary Figure S1).

Finally, here we compared the results of our method with three other tools that explore structural features for pocket classification and have shown to perform well in previous study (30): MaSIF-ligand (11), KRIPO (8) and ProBiS (9). Except for MaSIF-ligand, the other three methods have been shown to achieve top performance in a previous study (30). As KRIPO does not support fingerprints for the HEME ligand, this was removed from the comparison. Overall, CSM-Potential achieved ROC AUC of 0.96, which is comparable to MaSIF-ligand with 0.95, and superior to the performance of KRIPO and ProBiS with ROC AUC of 0.93 and 0.90, respectively (Figure 2B). A more recent version of the MaSIF framework has been proposed, namely dMaSIF (31), however we opted to not include it in our comparison given that we were unable to run it locally based on the instructions available.

## CONCLUSION

Here, we present CSM-Potential, a webserver that combines geometric deep learning with our graph based signatures for predicting likely binding regions based on protein surface. Our method has similar performance to state of the art methods with robust and accurate predictions on non-redundant blind test sets for identification of PPI binding sites and for the classification of biological ligands binding sites. We believe CSM-Potential will be of great value to the study of protein function prediction for both more experienced and also non-expert users. Our method is freely available as an easy-to-use webserver and API to facilitate large-scale processing and incorporation into analytical pipelines at http://biosig.unimelb.edu.au/csm_potential.

## DATA AVAILABILITY

CSM-Potential is freely available (no login or license required) as an easy-to-use webserver and API at http://biosig.unimelb.edu.au/csm_potential. Documentation on how to use the webserver and examples for querying the API using the Python programming language are available at http://biosig.unimelb.edu.au/csm_potential/help. Finally, all the experimental data used to train and evaluate the predictive models described in this work can be accessed at http://biosig.unimelb.edu.au/csm_potential/data.

## Supplementary Material

gkac381_Supplemental_File
