# Supplementary material for: CSM-Potential: mapping protein interactions and biological ligands in 3D space using geometric deep learning
Source: Nucleic Acids Res. 2022 May 24;50(W1):W204–9. doi: 10.1093/nar/gkac381 (PMC9252741; doi:10.1093/nar/gkac381)
Supplement: gkac381_Supplemental_File [file gkac381_supplemental_file.docx]

# SUPPLEMENTARY DATA

**CSM-potential: mapping PrOTEin iNteracTIons And biological Ligands in 3D space using geometric deep learning**

Carlos H. M. Rodrigues ^1,2,*^, David B. Ascher ^1,2,*^

^1^ Computational Biology and Clinical Informatics, Baker Heart and Diabetes Institute, Melbourne, Victoria, Australia

^2^ School of Chemistry and Molecular Biosciences, University of Queensland, Brisbane, Queensland, Australia

* To whom correspondence should be addressed. D. B. A. Tel: +61 90354794; Email: [d.ascher@uq.edu.au](mailto:d.ascher@uq.edu.au); C. H. M. R. Tel: +61 385321111; Email: [carlos.rodrigues@baker.edu.au](mailto:carlos.rodrigues@baker.edu.au)

#

# TABLES

**Table S1 - Summary of ligands present in the dataset used for biological ligand classification.**

| **Ligand** | **PubChem CID** | **Name** | **Molecular Weight** |
| --- | --- | --- | --- |
| ADP | 6022 | Adenosine diphosphate | 427.20 |
| CoA | 87642 | Coenzyme A | 767.50 |
| FAD | 643975 | Flavin adenine dinucleotide | 785.50 |
| HEM | 26945 | Heme | 616.50 |
| NAD | 5893 | Nicotinamide adenine dinucleotide | 664.40 |
| NADP | 5886 | Nicotinamide adenine dinucleotide phosphate | 744.40 |
| SAM | 34756 | S-adenosyl methionine | 399.40 |

**Table S2 - Performance during training of CSM-Potential for PPI binding site prediction.** Results are shown as mean values for each evaluation metric after 5 repetitions.

| **Method** | **AUC** | **MCC** | **F1** | **Sensitivity** | **Specificity** |
| --- | --- | --- | --- | --- | --- |
| CV1 (80/20) | 0.82 | 0.23 | 0.29 | 0.77 | 0.76 |
| CV2 (50/50) | 0.79 | 0.18 | 0.20 | 0.73 | 0.69 |

# FIGURES


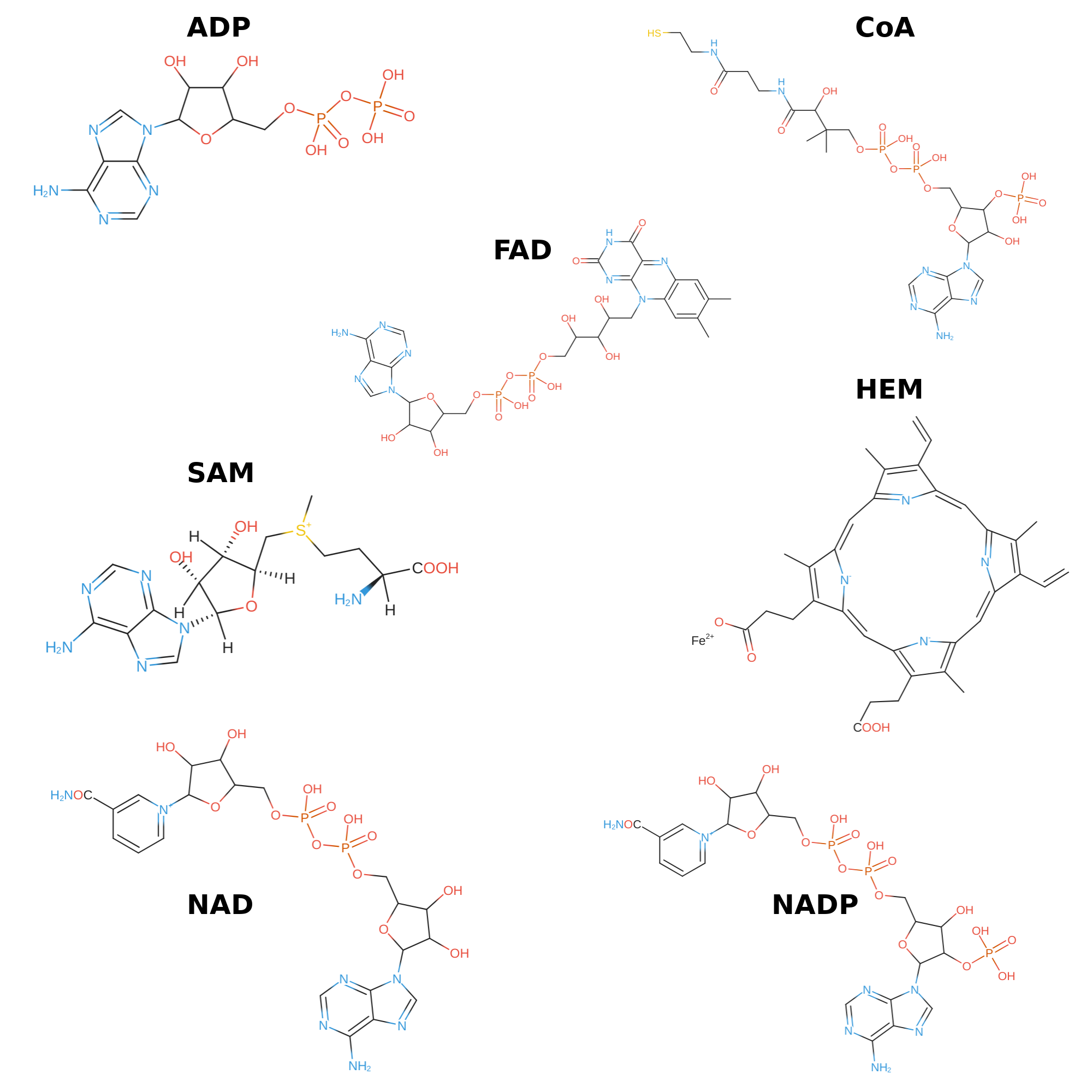


**Figure S1 - Chemical structural for all 7 ligands present in the dataset used for biological ligand classification.** Depictions were generated based on the canonical smiles available in the PubChem database^1^ and the SmilesDrawer tool^2^.


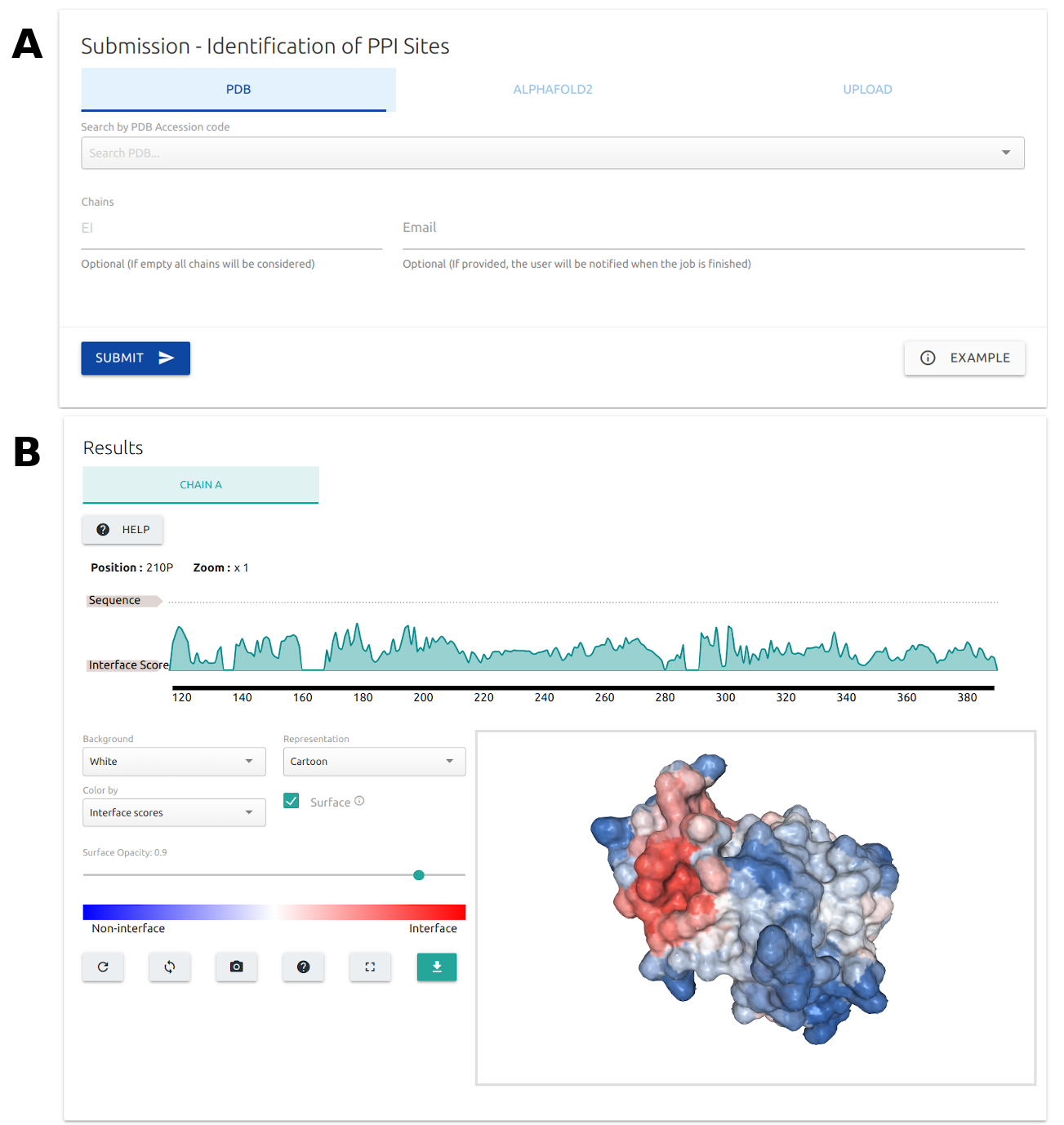


**Figure S2 - Graphical interface for submission and results page of CSM-Potential for predicting PPI binding sites.** Panel A shows a screenshot of the submission page, where users are required to provide a protein structure by input a valid accession code for structures on the Protein Data Bank, select from structures available in the AlphaFold database, or upload their own structure in PDB format. On the results page (Panel B), predicted scores for each residue are shown at a sequence level using the FeatureViewer component at the top. Predictions are also mapped on the input 3D structure and displayed in an interactive viewer using NGLviewer.


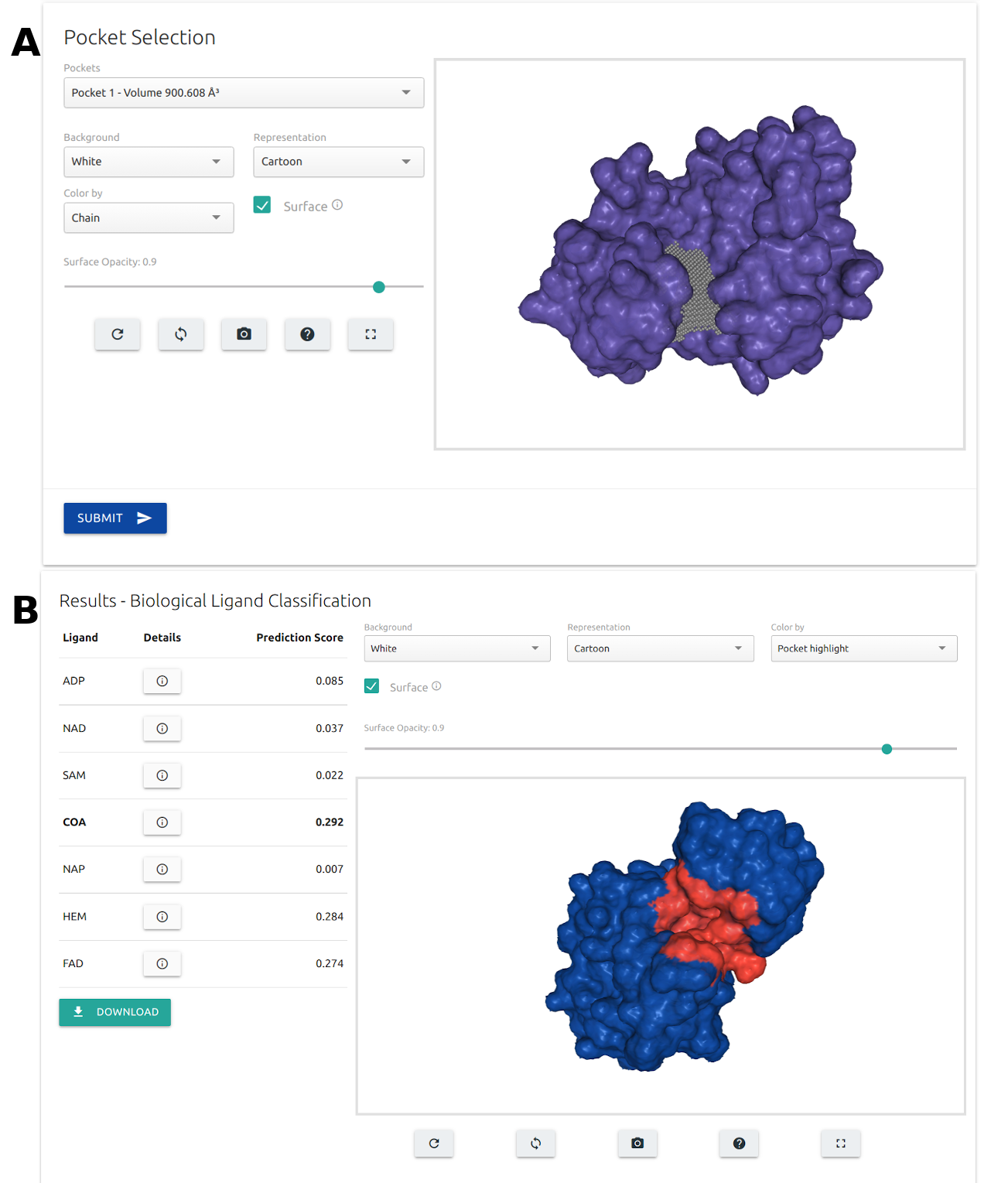


**Figure S3 - Graphical interface for submission and results page of CSM-Potential for the biological ligand classification.** Similarly to the PPI binding site predictions, users are also required to provide a structure by either providing a valid PDB accession code, selecting a model structure from the AlphaFold database, or uploading their own structures. However, for the biological ligand classification, one extra step is required in order to select the pocket region which will be analysed during the predictions (Panel A). Here a list of pockets identified via Ghecom are shown at the top of the page. Panel B displays the results page, where prediction scores for 7 different ligands are shown in a table format on the left, while the input structure with the option to highlight the selected pocket is shown on the right. For each ligand, a Details button is displayed, allowing users to analyse general chemical properties for a particular ligand, including molecular weight and their chemical structure depicted using the SmilesDrawer package.


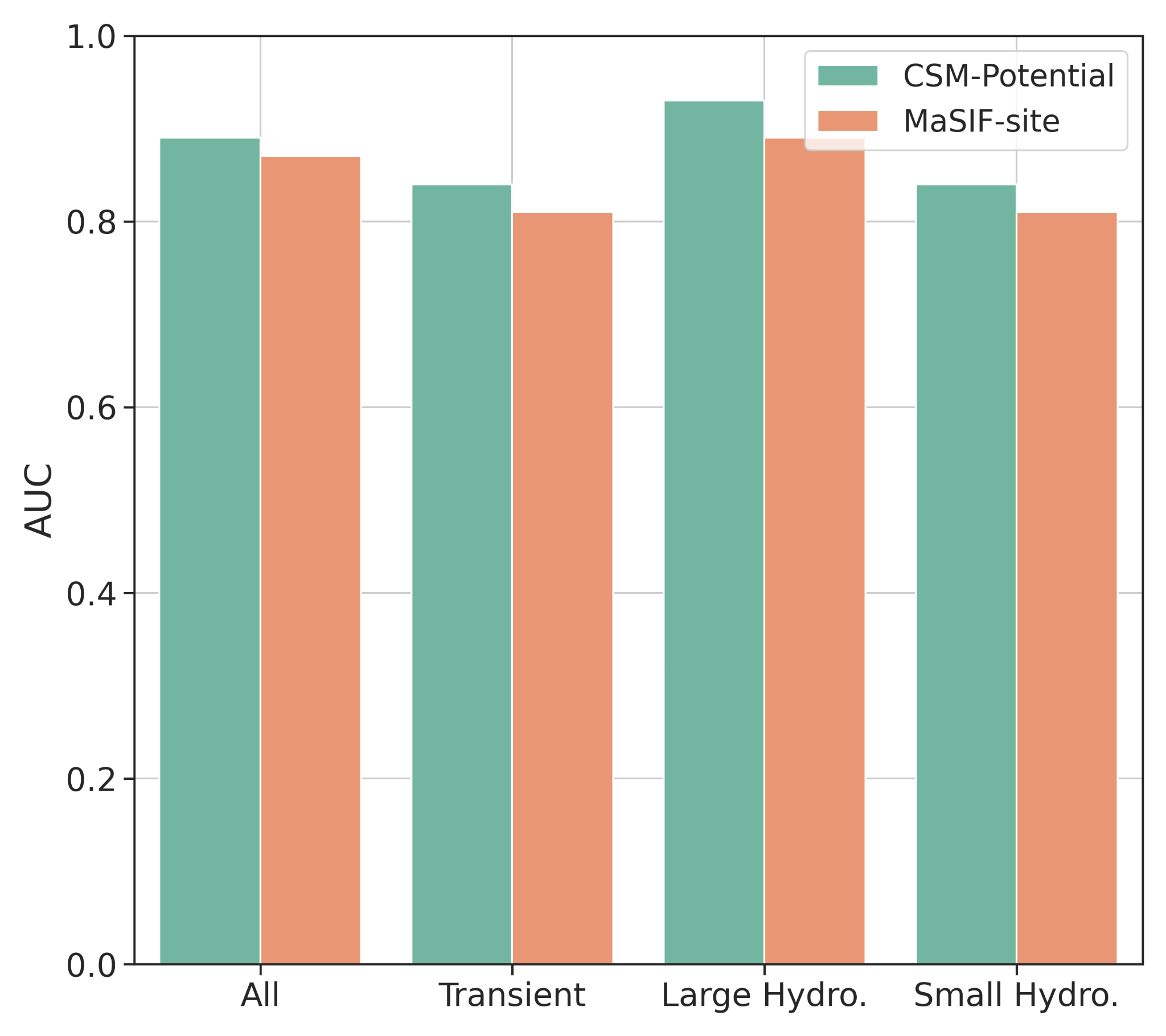


**Figure S4 - Performance comparison of CSM-Potential and MaSIF-site on a non-redundant blind-test set.** Results are presented as median ROC AUC per protein for (from left to right) all entries in the blind-test, only transient interactions, and PPIs with large and small hydrophobic interfaces.


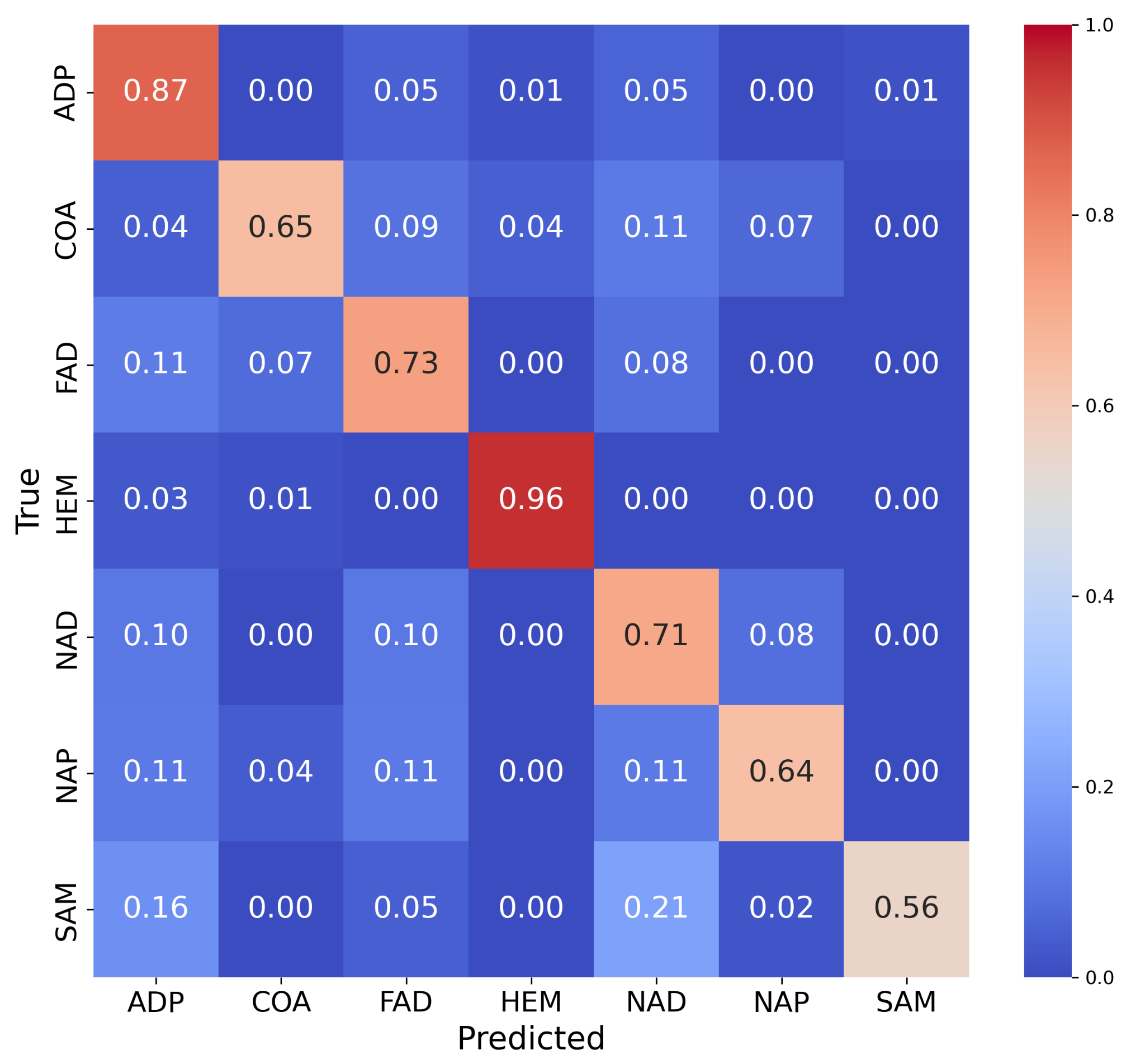


**Figure S5 - Confusion matrix for performance of CSM-Potential on a non-redundant blind-test set for biological ligand classification task.** Cells are coloured according to CSM-Potential performance for predictions on specific ligands where blue and red represent low and high predictive scores, respectively.

# REFERENCES

1. Kim, S., J. Chen, T. Cheng, et al., *Pubchem in 2021: New Data Content and Improved Web Interfaces.* Nucleic Acids Res, 2021. **49**(D1): p. D1388-D1395.

DOI: 10.1093/nar/gkaa971.

2. Probst, D. and J.L. Reymond, *Smilesdrawer: Parsing and Drawing Smiles-Encoded Molecular Structures Using Client-Side Javascript.* J Chem Inf Model, 2018. **58**(1): p. 1-7.

DOI: 10.1021/acs.jcim.7b00425.
